# Vaginal Ovariotomy

**Published:** 1870-10

**Authors:** P. G. Thomas


VAGINAL OVARIOTOMY. *By Dr. P. G. Thomas.*—In a multipera of sparn habit and nervous temperament, there was discovered a movable cyst behind the uterus which pushed that organ forward, and occupied completely the cul-de-sac of Douglas. It was of the size of a large orange, and could easily be pushed out of the pelvic cavity. The patient had supposed she had retroflexion, but Dr. J. L. Brown diagnosed it as cystic degeneration of the ovary.

On consultation three plans of procedure were proposed: that the cyst be allowed to develop, and be operated upon sometime in the future; that the cyst should be tapped per-vaginam; and that the operation of ovariotomy be performed through the fornix vaginæ, in the same manner that it is usually done, through the abdominal walls. The last proposition was made by myself, and upon these grounds:

1. The cyst being movable, sufficient space could be obtained through the fornix vaginaæ to withdraw the empty sac.

2. This procedure I preferred to simple tapping, which is liable to be followed by drainage and exhaustion to the patient. The increase of danger was not regarded as great, even if removal of the cyst proved impossible.

3. An early operation was advisable, owing to the anxious nature of the patient.

The operation being decided upon, the patient was etherised and placed in the knee-elbow position by the apparatus of Dr. Bozeman.

To prevent the rectum falling into the line of incision, a rectal bougie was inserted for about five inches.

Sims' speculum was introduced, the perineum and posterior vaginal wall were lifted, and the fornix vaginæ was caught midway between the cervix and rectum with a tenaculum, drawn well down, and with a pair of long scissors, one limb of which was placed against the rectum and the other against the cervix, a cut was made through it into the peritoneum at one stroke.

The patient's position was now changed to the dorsal decubitus, and, passing a finger through the incision, it touched the tumor which had fallen again into the pelvis.

With a trochar—it having been secured with a tenaculum—I punctured three cysts which gave vent to six or eight ounces of fluid, resembling vomited bile. Drawing upon the cyst it passed easily into the vagina.

The patient was now placed in Sims' position, on the left side, and his speculum introduced. A needle, armed with a strong double silk ligature, was passed through the pedicle at its point of exit through the vaginal wall, and each half of the penetrated tissue was tied, and both cyst and ligature were cut off. The cul-de-sac of Douglas was then sponged, the pedicle returned to the abdominal cavity, and the incision closed by one silver suture.

After the operation the patient was kept quiet by opium, and kept in the supine position. After ten days of improvement, and without any unfavorable symptoms, she was allowed to exercise herself unduly, and brought on an attack of peri-uterine cellulitis. She recovered from this, and on the thirtieth day was declared out of danger.

Any one undertaking this operation should rehearse the first step of it upon the cadaver before attempting it upon the living subject. Before making this operation, one attempt was made on the cadaver. The difficulty in this attempt induced me to practice the procedure on seven other dead bodies before attempting it on the living.—*Am. Journal Medical Science.*

